# Improving Assessment of the Spectrum of Reward-Related Eating: The RED-13

**DOI:** 10.3389/fpsyg.2017.00795

**Published:** 2017-05-30

**Authors:** Ashley E. Mason, Uku Vainik, Michael Acree, A. Janet Tomiyama, Alain Dagher, Elissa S. Epel, Frederick M. Hecht

**Affiliations:** ^1^UCSF Department of Psychiatry, Center for Health and Community, San FranciscoCA, United States; ^2^UCSF Osher Center for Integrative Medicine, San FranciscoCA, United States; ^3^Montreal Neurological Institute, McGill University, MontrealQC, Canada; ^4^Institute of Psychology, University of TartuTartu, Estonia; ^5^Department of Psychology, University of California, Los Angeles, Los AngelesCA, United States

**Keywords:** reward-related eating, obesity, assessment, eating behavior, uncontrolled eating, reward-driven eating, item response theory

## Abstract

A diversity of scales capture facets of reward-related eating (RRE). These scales assess food cravings, uncontrolled eating, addictive behavior, restrained eating, binge eating, and other eating behaviors. However, these scales differ in terms of the severity of RRE they capture. We sought to incorporate the items from existing scales to broaden the 9-item Reward-based Eating Drive scale (RED-9; Epel et al., 2014), which assesses three dimensions of RRE (lack of satiety, preoccupation with food, and lack of control over eating), in order to more comprehensively assess the entire spectrum of RRE. In a series of 4 studies, we used Item Response Theory models to consider candidate items to broaden the RED-9. Studies 1 and 2 evaluated the abilities of additional items from existing scales to increase the RED-9’s coverage across the spectrum of RRE. Study 3 evaluated candidate items identified in Studies 1 and 2 in a new sample to assess the extent to which they accounted for more variance in areas less well-covered by the RED-9. Study 4 tested the ability of the RED-13 to provide consistent coverage across the range of the RRE spectrum. The resultant RED-13 accounted for greater variability than the RED-9 by reducing gaps in coverage of RRE in middle-to-low ranges. Like the RED-9, the RED-13 was positively correlated with BMI. The RED-13 was also positively related to a diagnosis of type 2 diabetes as well as cravings for sweet and savory foods. In summary, the RED-13 is a brief self-report measure that broadly captures the spectrum of RRE and may be a useful tool for identifying individuals at risk for overweight or obesity.

## Introduction

Eating for pleasure is ubiquitous in the modern food environment. Easy access to highly palatable foods, especially those high in combinations of sugar, fat, and salt, constantly tempt individuals to eat for the rewarding experience of doing so, rather than for homeostatic caloric need (Lowe, 2003). Positive emotions, such as happiness and celebratory states, or negative emotions, such as stress or anxiety, can motivate such reward-related eating (RRE) so as to amplify (positive reinforcement) or reduce (negative reinforcement) the emotional state, respectively (Skinner, 1963). Repeated RRE of highly palatable foods in response emotional states can form the basis of habitual overeating that may precipitate eating pathology (e.g., binge eating disorder). Hence, researchers at intersections of health behavior, nutrition, and metabolic health, among others, often assess dimensions of RRE before, during, and after implementing interventions targeting health behavior change in the context of metabolic syndrome and its related conditions (e.g., O’Neil et al., 2012; Forman et al., 2013; Mason et al., 2016).

A plethora of scales gauge degrees of RRE by assessing various severities of food cravings, uncontrolled eating (UE), addictive behavior, restrained eating, binge eating, and other problematic eating behaviors (Price et al., 2015; Vainik et al., 2015b). These differ in terms of whether they focus on assessing problematic eating behavior at lower, middling, and higher levels on the continuum of overeating (e.g., Davis, 2013; Vainik et al., 2015b). For example, the Yale Food Addiction Scale (YFAS; Gearhardt et al., 2009, 2016) assesses eating behavior in terms of the Diagnostic and Statistical Manual of Mental Disorders (DSM) criteria for substance dependence. Thus, the YFAS likely assesses RRE at the severe end of the pathological overeating continuum. Similarly, the Binge Eating Scale (BES; Gormally et al., 1982) focuses on binge eating behavior, which is a more severe manifestation of problematic overeating. In contrast, the Palatable Eating Motives Scale (PEMS; Burgess et al., 2014) and the Power of Food Scale (Lowe et al., 2009) assess reasons for overeating behavior and the impact of the environment on eating-related choices, and thus seem to focus more on less severe levels of overeating.

A recent Item Response Theory (IRT) analysis of various eating-related scales (Vainik et al., 2015b) supports this perspective: Analyses indicated that different scales tend to best capture variability at different levels of UE (one dimension of RRE). For example, items assessing eating impulsivity (e.g., Vainik et al., 2015a) better assess lower levels of UE, items assessing emotional eating (e.g., emotional eating items of the Dutch Eating Behavior Questionnaire [DEBQ]; van Strien et al., 1986) better assess middle levels of UE, and items assessing binge eating (e.g., BES; Gormally et al., 1982) better assess higher levels of UE. These different aspects of eating may reflect developmental phases through which one develops problematic overeating pathology. For example, individuals who have greater eating-related impulsivity who then cultivate a habit of eating to cope with emotions may eventually develop chronic, uncontrolled binge eating (Davis, 2013). Taken together, both theoretical and empirical evidence suggest that no single one of these scales assesses the entirety of the RRE continuum directly and comprehensively. Thus, researchers must often combine measures to capture variability across the spectrum of RRE.

To address this issue, Epel et al. (2014) developed the 9-item Reward-based Eating Drive (RED-9) scale to assess the entire spectrum of RRE. The RED-9 correlates with BMI cross-sectionally and also predicts changes in BMI over time (Epel et al., 2014), and recent studies have shown that reductions in RRE as assessed by the RED-9 are a mechanism by which weight loss interventions impact weight change (Mason et al., 2016). Additionally, the RED-9 may index reward-related activity in the endogenous opioid pathway: In a sample of obese women, higher RED-9 scores were associated with greater daily craving intensity; however, on days when women received an opioidergic blockade, this association was not evident (Mason et al., 2015a).

Although the RED-9 scale is brief and simply worded, the extent to which it assesses the full spectrum of RRE pathology is uncertain. To date, no self-report scale has explicitly sought to assess the entire spectrum of RRE severity. A scale that assesses a broad spectrum of RRE severity would reduce the problems created by floor or ceiling effects that occur when, for example, the RED-9 is associated with an outcome only at a particular level of RRE severity.

### Purpose and Overview of Studies

In this series of studies, we sought to broaden the original 9-item RED-9 (Epel et al., 2014) to capture variability across the entire spectrum of RRE. The RED-9 assesses three constructs: lack of satiety, preoccupation with food, and lack of control over eating, and comprises both items derived from existing questionnaires, namely the BES (Gormally et al., 1982) and the Three Factor Eating Questionnaire (TFEQ; Stunkard and Messick, 1985), as well as newly developed items. We employed IRT (Baker, 2001; Partchev, 2004; Wirth and Edwards, 2007; Revelle, 2014) to improve the ability of RED-9 to capture RRE across the full spectrum of such eating behavior - that is, ranging from the lowest levels of eating for pleasure to the highest levels of pathological overeating. Studies 1 and 2 made use of existing data sets to examine additional items from existing scales as potential additions to the RED-9 that would allow it to cover more variability across the spectrum of RRE. Analyses from Studies 1 and 2 informed our original data collection for Studies 3 and 4: Study 3 evaluated the candidate items identified in Studies 1 and 2 in a new sample to assess the extent to which they accounted for more variance in areas that were less well-covered by the RED-9. Study 4 tested the ability of the revised 13-item RED scale (RED-13) to provide consistent coverage across the range of the RRE spectrum.

## Study 1

### Aim

Study 1 aimed to examine the extent to which items from existing measures of eating behavior would provide additional coverage of the RRE construct using two existing datasets collected from individuals of obese status to allow us to oversample individuals with overeating pathology who would endorse more severe items at a greater rate.

### Method

#### Participants

See **Table 1** for sample information. Participants were drawn from two previously conducted studies, the primary aims, recruitment details, and study design of which are described elsewhere (Sample 1: Daubenmier et al., 2016, *n* = 194; Sample 2: Mason et al., 2015a, *n* = 44).

**Table 1:** Participant characteristics for all studies.

Study	1	2	3	4
*N*	238	380	349	346
Age, *M* (*SD*)	44.25 (13.14)	32.98 (11.53)	34.23 (10.60)	35.43 (11.04)
BMI, *M* (*SD*)	35.29 (13.14)	28.07 (7.24)	26.40 (6.72)	25.65 (6.6)
Sex, *N* (%) Female	199 (83.6%)	171 (45.0%)	137 (39.3%)	167 (48.3%)
Race/Ethnicity, *N* (%)				
White	129 (54.2%)	280 (73.7%)	231 (66.2%)	243 (70.2%)
Black	39 (16.4%)	25 (6.8%)	15 (43.0%)	26 (7.5%)
Asian / Pacific Islander	26 (10.9%	21 (5.5%)	64 (18.3%)	41 (11.9%)
Hispanic / Latino	26 (10.9%)	20 (5.3%)	25 (7.2%)	25 (7.2%)
Native American / Alaska Native	2 (1.0%)	0 (0.0%)	2 (0.6%)	2 (0.6%)
Mixed Race	15 (6.3%)	9 (2.4%)	2 (0.6%)	9 (2.6%)
Declined Response	1 (0.4%)	25 (6.8%)	0 (0.0%)	0 (0.0%)
Education, *N* (%)				
Some High School	0 (0.0%)	ˆ	35 (10%)	1 (0.3%)
High School Diploma	25 (10.5%)	ˆ	77 (22.1%)	42 (12.1%)
Some College	ˆ	ˆ	ˆ	100 (28.9%)
Associates Degree (AS, AA)	26 (10.9%)	ˆ	45 (12.9%)	33 (9.5%)
Bachelor’s Degree (BA, BS)	132 (55.5%)	ˆ	150 (43.0%)	137 (39.6%)
Advanced Degree (MA, MS, MD, PhD, JD)	53 (22.3%)	ˆ	40 (11.5%)	32 (9.25%)
Declined Response	2 (0.8%)	ˆ	2 (0.6%)	1 (0.3%)


*ˆ indicates response option not present in this study.*

#### Procedures

The University of California, San Francisco Institutional Review Board (IRB) approved of all procedures and all participants provided written informed consent. All data for this study were collected at participants’ baseline visits. Participants in each abovementioned sample completed survey instruments in person during a baseline visit. In addition to completing the below-listed surveys, participants completed a survey of basic demographic information. A research assistant collected anthropometric measures, including height and weight.

#### Measures

All surveys were completed in person on a computer. Scales were administered through the Research Electronic Data Capture (RedCap) survey system (Harris et al., 2009).

#### Reward-Based Eating Drive Scale (RED-9; Epel et al., 2014)

The RED-9 assesses three dimensions of RRE: loss of control over eating, lack of satiety, and preoccupation with food. Of the 9 items, 2 items originate in the BES (Gormally et al., 1982), 4 items originate in the TFEQ (Stunkard and Messick, 1985), and 3 items were developed for this scale. Sample items include, “*When I start eating, I just can’t seem to stop*” (lack of control), “*I don’t get full easily*” (lack of satiety), and “*Food is always on my mind*” (preoccupation with food). In this study, participants answered on original scales: 3-point or 4-point scales for BES items, 2-point scale for TFEQ, and a 5-point scale for original items (1 = *strongly disagree* to 5 = *strongly agree*]. Total scores for this sample were computed by taking the z-scores of each item before averaging all items. Higher scores reflect higher RED.

#### Binge Eating Scale (BES; Gormally et al., 1982)

The 16-item BES assesses binge eating severity. Respondents endorse one of three statements (2 items) or four statements (14 items) for each item, and items are scored such that higher numbers indicate greater binge eating pathology. Total scores are computed as sums, with scores of 17 or lower generally indicating mild or no binge eating, 18–26 indicating moderate binge eating, and 27 or greater indicating severe binge eating.

#### Yale Food Addiction Scale (YFAS; Gearhardt et al., 2009)

The 25-item YFAS assesses pathological levels of food addiction symptoms based on the 7 symptoms of substance dependence articulated in the *DSM-IV-TR* (e.g., withdrawal, tolerance, continued use despite problems; American Psychological Association, 2000). Participants respond on scoring schemes that include dichotomous and frequency scoring (e.g., ranging from *Never* to *Four or more times daily*). A total summed YFAS score was computed using the continuous summed score method of dichotomous items (three items are ‘primer’ items and not intended to be included in the total score; e.g., Price et al., 2015), as well as a total symptom count method, where total scores range from 0 (*0 symptoms of food addiction*) to 7 (*7 symptoms of food addiction*).

#### Dutch Eating Behavior Questionnaire (DEBQ; van Strien et al., 1986)

This 33-item scale comprises three subscales. The 10-item Restraint subscale (DEBQ-R) assesses dietary restraint, which has also been termed cognitive restraint. The 10-item External Eating subscale (DEBQ-X) assesses the tendency to eat in response to external food-related cues such as the sight, taste, and smell of attractive food. The 13-item Emotional Eating subscale (DEBQ-E) assesses eating triggered by specific and diffuse emotions such as anger, boredom, anxiety, or fear. Participants respond to items on a scale from 1 (*never*) to 5 (*very often*). In this study, participants completed only the DEBQ-E. The total subscale score was computed as the sum of the 13 items.

#### Three Factor Eating Questionnaire (TFEQ; Stunkard and Messick, 1985)

The 51-item TFEQ comprises three subscales. The 20-item cognitive restraint subscale (TFEQ-R) assesses conscious mechanisms for restraining food intake. The 20-item disinhibition subscale (TFEQ-D) assesses the extent to which one feels that he or she cannot control his or her eating. The 15-item hunger subscale (TFEQ-H) assesses feelings of hunger and its behavioral consequences. Participants select true or false for 36 items, rate 13 items on a scale from 1 (*rarely*) to 4 (*always*), rate 1 item on a scale from 0 (*eat whatever I want, whenever I want it*) to 5 (*constantly limiting food intake, never giving in*), and 1 item on a scale from 1 (*not like me*) to 4 (*describes me perfectly*). Subscale scores are summed, with higher scores indicating greater pathology (e.g., higher dietary restraint, disinhibition, and hunger). As in published literature using this measure (e.g., French et al., 2014), we independently examined subscales.

#### Demographics and Anthropometrics

Participants indicated their age, biological sex, educational attainment, race/ethnicity, and total annual household income. Trained research assistants measured participants’ weight and height, with which we computed body mass index (BMI).

#### Analytic Plan

First, we examined the extent to which the RED-9 correlated with each of the other scales by comparing the total scores using bivariate correlations. We next examined the extent to which each of these scales correlated with BMI after log transforming BMI to adjust for normality and residualizing for demographic covariates (age, education, race/ethnicity, income, and biological sex). Second, we conducted a confirmatory factor analysis (CFA) of the RED-9 to test the scale’s unidimensionality. We assessed model fit using typical fit criteria {Comparative Fit Index [CFI] > 0.95, Root Mean Square Error of Approximation [RMSEA] < 0.06, Standardized Root Mean Square Residual [SRMR] < 0.08 (Hu and Bentler, 1999; Kenny, 2014)}. Third, we used separate CFA models to assess whether each item from the other scales would be suitable as a tenth item in the RED-9. We retained items with factor loadings greater than or equal to 0.45, an acceptable and reasonable cut-off (Hair et al., 1998) that allowed analyses to retain items that explain considerable variability in, and are strongly related to, RRE.

Fourth, we built a final CFA model based on all retained candidate items, as well as all RED-9 items, and used IRT to analyze this larger model. A typical 2-parameter IRT model considers both discrimination/factor loading and severity of an item (Revelle, 2014). As we had already built a unidimensional model where all items had reasonable factor loadings, we only focused on the severity of the items, which makes the analysis similar to a 1-parameter IRT model, (see Baker, 2001; Partchev, 2004; Wirth and Edwards, 2007; Revelle, 2014; Vainik et al., 2015a) for accessible reviews. Item severity refers to the locations of item thresholds – the value on the latent continuum where the probability of endorsing “this level or higher” response option is 50%. For example, for a 5-point response scale, where options are labeled from “1” to “5,” the first threshold is the point on the latent continuum where there is a 50% probability of endorsing the second or higher response option. The number of thresholds for an item depends on number of response options. An item with *k* response options has *k-*1 thresholds. The average of an item’s threshold location parameter is often termed its “difficulty,” as IRT was first applied in aptitude tests. Here, “severity” is used as a more suitable descriptor in current context.

The goal of this analysis is to ascertain whether item thresholds are distributed across the whole latent continuum of the trait (in this case, RRE). We identified considerable gaps where the distance between two thresholds was wider than 0.29 normal units. We derived this gap size from a logistic model based on IRT, as modeling a gap of 0.50 logit units is often considered clinically significant (Lai and Eton, 2002). We posited that the criterion of 0.50 logit units is a reasonable tradeoff between threshold density and scale brevity. We then converted logit units to normal units by dividing by a factor of 1.7 (0.5/1.7 = 0.29), as normal models are simply scaled from logit models by a constant of 1.7 (Camilli, 1994), and our analysis package provides normal units. After we identified gaps, we then retained all items that provided coverage in at least one gap left by the RED-9 for the next analysis.

We conducted all analyses in R 3.32 (R Core Team, 2013). We built all factor models with the “lavaan” package version 0.5–22 (Rosseel, 2012), treated items as categorical variables using the WLSMV estimator, and used pairwise deletion for missing values. We extracted thresholds from fitted objects using lavaan’s inspect (fit, what = “th”) command. We used ggplot2, RColorBrewer, and GGthemes to create figures (Wickham, 2009; Arnold, 2013; Neuwirth, 2014).

### Results

#### Correlations

As shown in **Table 2**, the RED-9 was highly correlated with each scale, except for the TFEQ-R, as it assesses a conceptually different construct (restraint). The correlation between the RED-9 and BMI was relatively low; however, this may be due to the BMI range being restricted to 30 or greater in this sample.

**Table 2:** Correlations among the RED-9 and other scales in Study 1.

Scale	RED-9	BES	DEBQ-E	YFAS-SUM	YFAS-SXS	TFEQ-R	TFEQ-D	TFEQ-H
Mean (*SD*)	0 *(0.63)*	15.59 *(7.72)*	40.89 *(11.97)*	6.42 *(4.92)*	2.96 *(1.59)*	9.26 * (4.09)*	10.01 *(3.28)*	7.20 *(3.56)*
BES	0.68							
DEBQ-E	0.46	0.58						
YFAS-SUM	0.36	0.65	0.52					
YFAS-SXS	0.33	0.54	0.44	0.82				
TFEQ-R	-0.09	-0.14	-0.09	0.01	0.03			
TFEQ-D	0.69	0.68	0.63	0.49	0.44	-0.04		
TFEQ-H	0.65	0.62	0.45	0.46	0.44	-0.22	0.56	
BMI-RES	0.12	0.15	0.19	0.12	0.03	-0.07	0.17	0.12


*RED-9 = 9-item RED scale; BES = Binge Eating Scale; DEBQ-E = Dutch Eating Behavior Questionnaire – Emotional eating subscale; YFAS = Yale Food Addiction Scale: -Sum = Summed score, -SXS = Symptom count; TFEQ = Three Factor Eating Questionnaire: -R = Restraint, -D = Disinhibition, -H = Hunger; BMI-RES = Correlation residualized for Body Mass Index. BMI was log transformed and then residualized for age, education, race/ethnicity, income, and biological sex. Correlations or *r* = 0.19 or greater are statistically significant at *p* < 0.01; correlations of *r* = 0.15 or greater are statistically significant at *p* < 0.05.*

#### Confirmatory Factor Analysis (CFA)

**FIGURE 1:**
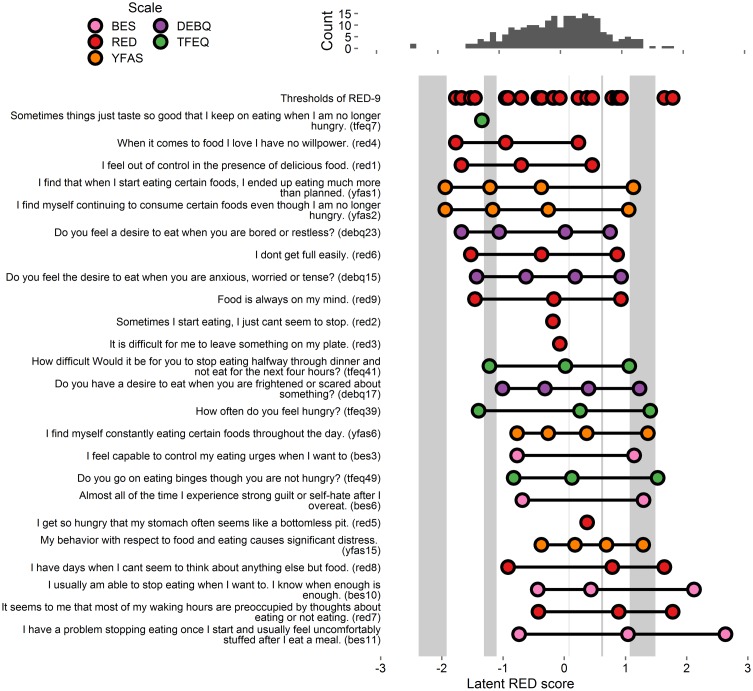
**Person-item map and histogram depicting thresholds on the spectrum of reward-related eating (RRE) for Study 1.** The histogram at top displays the locations of participants on the latent RRE construct. The top row of the person-item map at bottom depicts the locations of the gaps in coverage of the RRE construct left by the RED-9. The 27 items listed below “thresholds of RED items” are ordered by the average of their thresholds’ values, and colored by the respective scale from which they originate. Circles depict each threshold, i.e., location on the RRE construct where people are most likely to move from one response option to the next. Gray rectangles appear whenever the gap between two consecutive RED-9 item thresholds is wider than 0.29 units. The gray rectangle only highlights parts of the latent trait that are further than 0.29/2 units from any RED-9 threshold. This figure includes the RED-9 items and all items that account for variance in the gap areas. See **Supplementary Figure S1** for a figure with all items tested.

The RED-9 model’s suboptimal fit (1-factor model: *X*^2^ = 154.245, df = 27, *p* < 0.001, CFI = 0.928, RMSEA = 0.141, SRMR = 0.104) may have been due to some items having 2 response options instead of 5. For instance, see **Figure 1** for RED-9 items with just 1 threshold (described in Mason et al., 2015a, 2016). The fit improved with a 3-factor solution (3-factor model: *X*^2^ = 107.018, df = 24, *p* < 0.001, CFI = 0.953, RMSEA = 0.121, SRMR = 0.087), with the factors being highly correlated (factor correlations range: *r* = 0.72 to *r* = 0.92). The RED-9 items loaded onto each of three domains (loss of control over eating, lack of satiety, and preoccupation with food), as defined in Epel et al.’s (2014) original RED-9 validation paper. Thus, the 3-factor solution is an optimal fit to the data, and yields three distinct, yet highly correlated, subscales. At the same time, since the RED-9 is commonly treated as a single dimension scale (and scored as a summed total), we conducted the following item severity analysis using a unidimensional model.

We considered each of the items from the above-listed scales (BES, TFEQ, DEBQ, and YFAS) as potential additions to the RED-9. After removing duplicates with the existing RED-9 scale (5 items) we tested the remaining items (100) as potential suitable additions (per 0.45 loading criteria) to the RED-9 model. We computed 100 CFA models, with each model adding one of the 100 items to the RED-9. Of the items tested, 27 evidenced factor loadings above 0.45 (**Supplementary Figure S1**). We therefore retained these items for our third model. This third model (RED-9 plus 27 items) evidenced similar fit statistics to the RED-9 model (*X*^2^ = 1252.327, df = 594, *p* < 0.001, CFI = 0.905, RMSEA = 0.068, SRMR = 0.101).

#### Item Severity

Extracted thresholds from the final model appear in **Figure 1** and **Supplementary Figure S1**. Light gray boxes on the person-item map indicate the five gap areas in the RED-9’s coverage of the RRE construct. Of the 27 included items, 15 items accounted for variance in these gaps and were retained for analysis in Study 3 (these 15 items and all RED-9 items appear in **Figure 1**, all tested items appear in **Supplementary Figure S1**).

## Study 2

### Aim

Study 2 aimed to examine the extent to which items from existing measures of eating behavior would provide additional coverage of the RRE construct in a population-based sample accessed online.

### Methods

#### Participants

See **Table 1** for sample information.

#### Procedures

Participants learned of and participated in this study’s online survey study on the web-based Amazon Mechanical Turk (MTurk) platform (Buhrmester et al., 2011). Each MTurk respondent received $1.25 for questionnaire completion. The University of California, San Francisco IRB approved all study procedures. We used standard procedures to increase MTurk data reliability, which include excluding participants who incorrectly answer quality control questions designed to identify participants who respond without reading questions (Kittur et al., 2008).

#### Measures

Participants completed the TFEQ, the DEBQ, and demographic items described in Study 1, in addition to providing their height and weight. Additionally, participants completed the RED-9 as described in Study 1, except that all scale items were responded to on a scale from 0 (*strongly disagree*) to 5 (*strongly agree*). Participants also completed the following scales.

#### Palatable Eating Motives Scale (PEMS; Burgess et al., 2014)

The 19-item PEMS assesses four motives for eating tasty food (social, conformity, enhancement, and coping motives) and is modeled after the Drinking Motives Questionnaire (Cooper, 1994). Each subscale has 5 items, except the coping subscale, which has 4 items. Items are answered on a 5-point scale (*almost never/never, some of the time, half of the time, most of the time, almost always/always*). Total scores for each subscale are computed as the mean of all items for that subscale, with higher scores indicating greater eating of tasty food for that motive.

#### Food Craving Questionnaire – Trait – Reduced (FCQ-T-R; Meule et al., 2014)

The 15-item FCQ-T-R assesses (1) preoccupation with food, i.e., obsessive thoughts about food and eating, (2) loss of control over eating, i.e., difficulty regulating eating behavior when exposed to food cues, (3) positive outcome expectancy, i.e., believing that eating is positively reinforcing, and (4) emotional craving, i.e., the tendency to crave food when experiencing high levels of emotion. Items are answered on a 6-point scale from 1 (*never*) to 6 (*always*). In this study, all items were responded to on a scale from 0 (*strongly disagree*) to 5 (*strongly agree*). A total score is computed as the sum of all items.

#### Power of Food Scale (PFS; Lowe et al., 2009)

The 15-item PFS assesses the psychological impact of living in food-abundant environments by assessing appetite for highly palatable foods. Items are answered on a 5-point scale from 1 (*don’t agree at all*) to 5 (*strongly agree*). Items are averaged to compute a total scale score.

#### Analytic Plan

We conducted analysis in an identical fashion to Study 1.

### Results

#### Correlations

As shown in **Table 3**, the RED-9 was highly correlated with each scale, except the TFEQ-R, as in Study 1. The RED-9 was more highly correlated with BMI in this sample (relative to that of Study 1), likely due to the inclusion of individuals across all levels of BMI in this sample (not solely BMI > 30, as in Study 1).

**Table 3:** Correlations among the RED-9 and other scales in Study 2.

Scale	RED-9	PFS	FCQ-T-R	PEMS	TFEQ-R	TFEQ-D	TFEQ-H	DEBQ-R	DEBQ-E	DEBQ-X
Mean (*SD*)	0.0 *(0.77)*	42.80 *(14.22)*	42.65 *(19.38)*	41.87 *(15.93)*	9.59 *(5.10)*	7.43 *(4.08)*	6.99 *(4.22)*	26.78 *(8.82)*	33.42 *(14.88)*	31.30 *(7.60)*
PFS	0.76	–	–	–	–	–	–	–	–	–
FCQ-T-R	0.81	0.81	–	–	–	–	–	–	–	–
PEMS	0.57	0.67	0.68	–	–	–	–	–	–	–
TFEQ-R	-0.03	-0.09	0.01	-0.02	–	–	–	–	–	–
TFEQ-D	0.73	0.68	0.83	0.61	0.02	–	–	–	–	–
TFEQ-H	0.72	0.70	0.74	0.58	-0.15	0.72	–	–	–	–
DEBQ-R	0.21	0.15	0.27	0.21	0.79	0.29	0.09	–	–	–
DEBQ-E	0.65	0.68	0.82	0.69	-0.03	0.80	0.62	0.23	–	–
DEBQ-X	0.66	0.77	0.77	0.66	-0.14	0.72	0.71	0.12	0.72	–
BMI-RES	0.40	0.31	0.43	0.26	0.02	0.50	0.33	0.19	0.38	0.31


*DEBQ = Dutch Eating Behavior Questionnaire: -R = Restraint; -E = Emotional Eating; -X = Externalized Eating; PFS = Power of Food Scale; FCQ-T-R = Food Craving Questionnaire, Trait, Reduced; PEMS = Palatable Eating Motives Scale. See **Table 2** note for other abbreviations. Correlations of *r* = 0.12 or greater are statistically significant at *p* < 0.05. BMI was log transformed and residualized in an identical fashion to Study 1.*

#### Confirmatory Factor Analysis (CFA)

The RED-9 model demonstrated better fit indices in this analysis than in Study 1 (1-factor model: X^2^ = 414.835, df = 27, *p* < 0.001, CFI = 0.977, RMSEA = 0.195, SRMR = 0.068). The improved fit likely owes to all RED-9 items having five response options, which provided increased variance in responses and therefore allowed better modeling. Similar to Study 1, the model fit (specifically, RMSEA) improved with a 3-factor solution (3-factor model: X^2^ = 71.981, df = 24, *p* < 0.001, CFI = 0.997, RMSEA = 0.073, SRMR = 0.027) and the factors were highly correlated (factor correlations range: *r* = 0.79 to *r* = 0.81). A second unidimensional CFA that separately tested the fit of 132 additional items to the RED-9 indicated that 77 items evidenced factor loadings above 0.45 (**Supplementary Figure S2**). We therefore retained these items for our final model. This final model (77 items plus all RED-9 items) evidenced similar fit statistics to the RED-9 model (X^2^ = 9964.752, df = 3569, *p* < 0.001, CFI = 0.96, RMSEA = 0.069, SRMR = 0.09).

#### Item Severity

**FIGURE 2:**
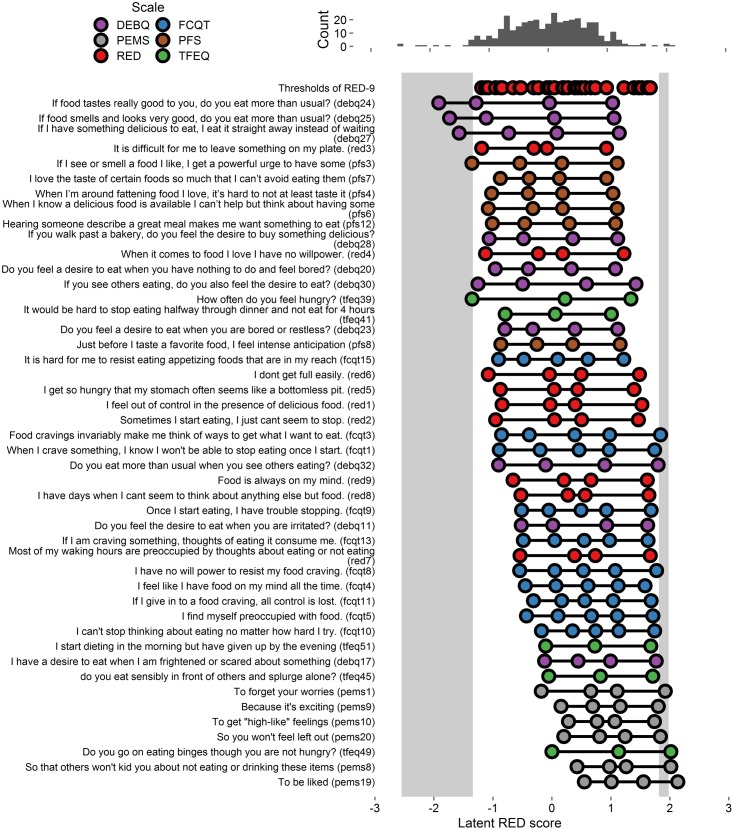
**Person-item map and histogram depicting thresholds on the spectrum of RRE for Study 2.** See **Figure 1** note. Some items are shortened for graphical presentation. This figure includes the RED-9 items and the 37 items that account for variance in the gap areas. See **Supplementary Figure S2** for a figure with all items tested.

Extracted thresholds appear in **Figure 2** and **Supplementary Figure S2**. As shown, the RED-9 provided better coverage of the RRE construct in Study 2 than it did in Study 1, as there are fewer and narrower light gray areas, indicating fewer and smaller gaps in coverage. Of the 77 added items, 37 items accounted for variance in the gap areas and were retained for Study 3 (these 37 items and all RED-9 items appear in **Figure 2**, and all tested items appear in **Supplementary Figure S2**).

## Study 3

### Aim

Study 3 evaluated the candidate items identified in Studies 1 and 2 in a new sample accessed online, using a 5-point Likert response scale for all items, to assess the extent to which they accounted for more variance in areas that were less well-covered by the RED-9.

### Methods

#### Participants

See **Table 1** for sample information.

#### Procedures

Procedures were identical to those used in Study 2, above. The University of California, San Francisco IRB approved of all procedures, and all participants provided written informed consent.

#### Measures

All questionnaire items were responded to on a scale from 0 (*strongly disagree*) to 4 (*strongly agree*).

#### Eating Impulsivity (EI; Vainik et al., 2015b)

We adapted two items that have previously been shown to target the lower extreme of the RRE construct. These items are, “*I tend to eat too much of my favorite food*” and “*sometimes I eat so much that I feel sick*.” Participants respond to items on a scale from 0 (*strongly disagree*) to 4 (*strongly agree*).

#### Analytic Plan

We (first three authors) collated all retained (non-RED) items from Studies 1 (*n* = 15) and 2 (*n* = 37) and independently categorized each item as assessing one of the three constructs captured by the RED-9 (lack of control over eating, lack of satiety, and preoccupation with food), or as not assessing any of these constructs. We then assessed interrater reliability using kappa coefficients (Fleiss, 1971). A fourth author resolved discrepancies, and we removed items falling outside these domains from consideration. We then conducted CFA and analyses as in Studies 1 and 2.

### Results

#### Item Selection

Of the items from Studies 1 and 2 (52 total), there were 5 overlaps, which resulted in 47 items for consideration. The three raters achieved high interrater reliability (kappa = 0.992), initially having disagreed on the categorization of 3 items, which were easily resolved after consulting with co-authors. Of the 47 items, authors agreed that 15 fell outside the three defining constructs of RRE captured by the RED-9 scale, which resulted in a total of 32 items for analysis.

#### Confirmatory Factor Analysis (CFA)

For comparison with Studies 1 and 2, we computed a CFA using the RED-9 (1-factor model: X^2^ = 264.551, df = 27, *p* < 0.001, CFI = 0.977, RMSEA = 0.159, SRMR = 0.059; 3-factor model: X^2^ = 48.272, df = 24, *p* = 0.002, CFI = 0.998, RMSEA = 0.054, SRMR = 0.027, factor correlations range: *r* = 0.79–0.92). We then computed a CFA that added the above-mentioned 32 additional items to the RED-9 (**Supplementary Figure S3**), and this model demonstrated similar fit statistics to the RED-9 model (X^2^ = 2738.463, df = 779, *p* < 0.001, CFI = 0.968, RMSEA = 0.085, SRMR = 0.076). We proceeded to extract thresholds using this model.

#### Item Severity

**FIGURE 3:**
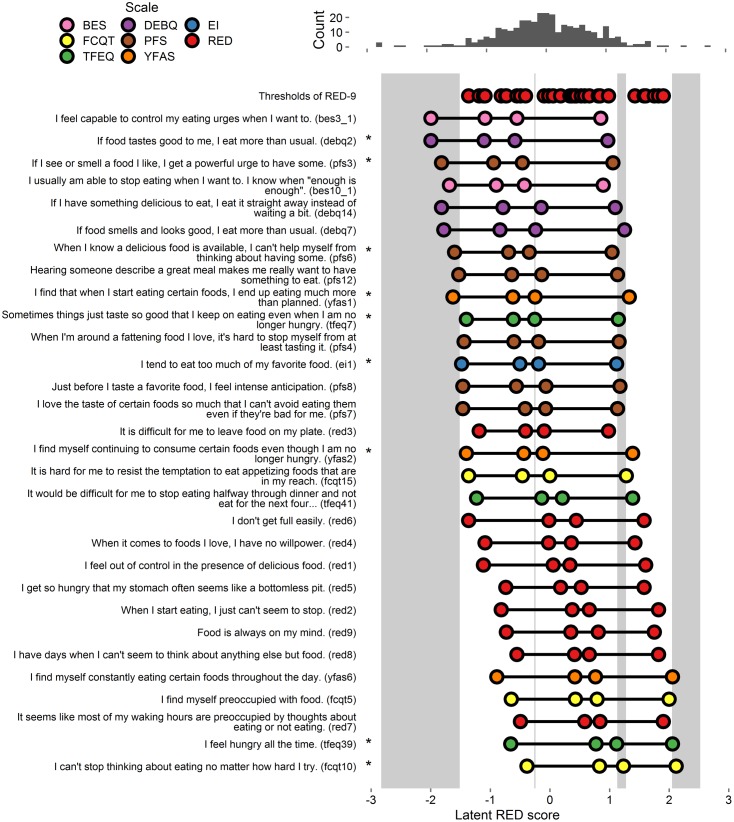
**Person-item map and histogram depicting thresholds on the spectrum of RRE for Study 3.** See **Figure 1** note. This figure includes the RED-9 items the 21 items that account for variance in the gap areas. For a graph on all items tested, see **Supplementary Figure S3**. Stars indicate items selected by authors for inclusion in Study 4.

Extracted thresholds appear in **Figure 3** and **Supplementary Figure S3**. As shown, there were gaps in coverage at each extreme, as well as gaps in the middle regions near 0 and 1. Of the 32 items, 21 provided coverage in one or more gaps. The first two authors independently selected items that, in total, provided coverage in all gaps. The authors both selected six items (DEBQ2, PFS3, PFS6, EI1, TFEQ39, and FCQ-T-R10), and one of the authors also selected an additional three items (YFAS1, YFAS2, and TFEQ7). These 9 items were retained for analysis in Study 4 (these 9 items and all RED-9 items appear in **Figure 3**, all tested items appear in **Supplementary Figure S3**).

## Study 4

### Aim

Study 4 evaluated the resultant items from Study 3 in a new sample accessed online to assess whether this new combination of items would improve upon the RED-9’s coverage of RRE by evaluating a revised scale (derived of analyses following from Study 3). In exploratory analyses, we also examined how the RED-13 relates to BMI, as reported in the RED-9 validation article (Epel et al., 2014). A diagnosis of type 2 diabetes, which can result from overeating of highly palatable foods, has previously been linked with eating impulsivity (Čukić et al., 2016) and self-reported cravings for savory and sweet foods, which correlate with both actual eating behavior and BMI (Boswell and Kober, 2016).

### Methods

#### Participants

See **Table 1** for sample information. The University of California, San Francisco IRB approved of all procedures, and all participants provided written informed consent.

#### Procedures

Procedures were identical to those used in Study 2, above. In addition to collecting BMI information, we also collected participants’ responses to measures of cravings for sweet and savory foods as well as their diabetes status (None, Type 1 or Type 2). Nineteen participants (5.49%) reported having Type 2 (T2) diabetes.

#### Measures

All survey items were responded to on a scale from 0 (*strongly disagree*) to 4 (*strongly agree*). Participants also reported on their diabetes status (Type 1 or Type 2).

#### Control of Eating Questionnaire (CoEQ; Dalton et al., 2015)

Of the 21 items in the CoEQ, we used the two subscales that specifically tap craving-related eating: One 4-item subscale assesses cravings for sweet foods and another 4-item subscale assesses cravings for savory foods. Representative items are, “*How often have you had cravings for sweet foods (cakes, pastries, biscuits, etc.)*?” and “*How often have you had cravings for starchy foods (bread, pasta)?*” Items are answered on a visual analog scale (values from 1 to 100) with anchors that go from *not at all strong*/*not at all* to *extremely strong*/*extremely often*. Total scores for each subscale are computed as the mean of items for that scale, with higher scores indicating stronger/greater craving.

#### Analytic Plan

We retained items resulting from Study 3 analyses (*n* = 9) and, together with all RED-9 items, conducted CFA and 1PL IRT model analyses as in Studies 1, 2, and 3. We then computed bivariate correlations between the resultant RED scale sum-score and each BMI, cravings for sweet, and cravings for savory. We also computed logistic regressions predicting a diagnosis of T2 diabetes (dichotomous variable) from resultant RED scale scores.

### Results

#### Confirmatory Factor Analysis (CFA)

The RED-9 model demonstrated a similar fit to those observed in Studies 1, 2, and 3 (1-factor model: X^2^ = 345.316, df = 27, *p* < 0.001, CFI = 0.967, RMSEA = 0.185, SRMR = 0.074; see **Table 4**). As in Studies 1, 2, and 3, model fit improved with a 3-factor solution (3-factor model: X^2^ = 60.022, df = 24, *p* < 0.001, CFI = 0.996, RMSEA = 0.066, SRMR = 0.029, factor correlations range: *r* = 0.75–0.81). We used an 18-item model (9 items plus all RED-9 items; X^2^ = 1225.077, df = 135, *p* < 0.001, CFI = 0.953, RMSEA = 0.153, SRMR = 0.1) for the basis of the item severity analysis.

**Table 4:** Factor loadings for items tested in Studies 3 and 4.

			Study 3				Study 4			
				
Item	Origin	# on RED Scale	Single-Factor	LOC	PO	LOS	Single-Factor	LOC	PO	LOS
I feel out of control in the presence of delicious food	Original	1	0.84	0.87			0.82	0.85		
When I start eating, I just can’t seem to stop	TFEQ 15	2	0.87	0.92			0.81	0.88		
It is difficult for me to leave food on my plate	TFEQ 16	3	0.65	0.67			0.61	0.64		
When it comes to foods I love, I have no willpower	Original	4	0.80	0.83			0.86	0.88		
I get so hungry that my stomach often seems like a bottomless pit	TFEQ 24	5	0.76		0.79		0.72		0.78	
I don’t get full easily	Original	6	0.61		0.63		0.59		0.65	
It seems like most of my waking hours are preoccupied by thoughts about eating or not eating	BES	7	0.86			0.90	0.88			0.91
I have days when I can’t seem to think about anything else but food	BES	8	0.83			0.88	0.80			0.84
Food is always on my mind	Original	9	0.83			0.87	0.88			0.91
*I feel hungry all the time*	*TFEQ 39*	*10*	0.80		0.83		0.81		0.89	
*I can’t stop thinking about eating no matter how hard I try*	*FCQT 10*	*11*	0.85			0.89	0.83			0.88
*I find myself continuing to consume certain foods even though I am no longer hungry*	*YFAS 2*	*12*	0.76	0.80			0.73	0.78		
*If food tastes good to me, I eat more than usual*	*DEBQ 2*	*13*	0.65	0.68			0.62	0.67		


*Items in italics are items added to the RED-9 to comprise the RED-13. Origin = Scale of item origin; RED label = Number of item on RED Scale; Single-Factor = Loading onto single-factor solution; TFEQ = Three Factor Eating Questionnaire; BES = Binge Eating Scale; Original = Original item; LOC = Loss of control; PO = Preoccupation with food; LOS = Lack of Satiety.*

#### Item Severity

**FIGURE 4:**
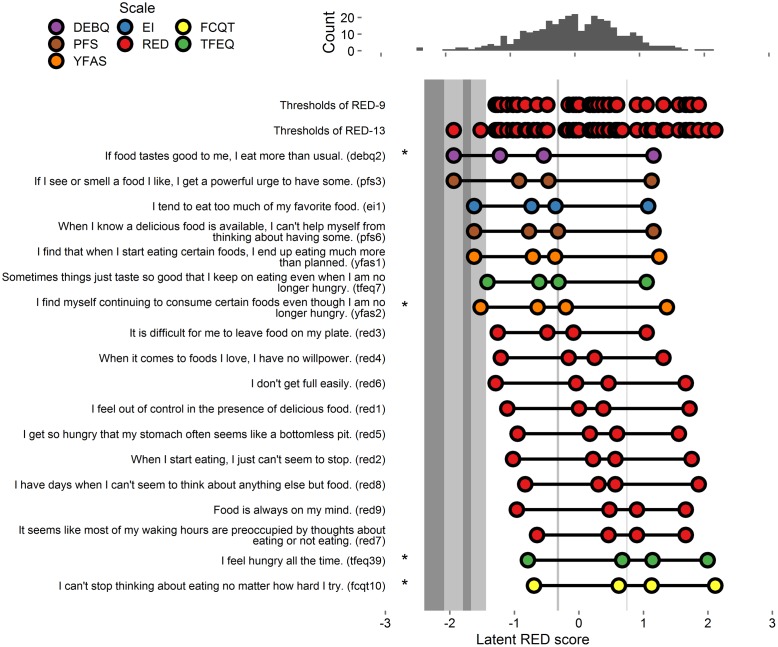
**Person-item map and histogram depicting thresholds on the spectrum of RRE for Study 4.** See **Figure 1** note. Bars in darker gray indicate gaps in RRE coverage left by the RED-13. Bars in lighter gray indicate gaps in coverage left by RED-9. Stars indicate items added to the RED-9 to form the RED-13.

Thresholds extracted from the full 18-item model (9 tested items and all RED-9 items) appear in **Figure 4**.

#### Final Scale Items

Of the candidate 9 items tested, 1 fell into the domain of lack of satiety, 2 fell into the domain of preoccupation with food, and 6 fell into the domain of loss of control over eating. To maximize coverage of the three domains, we first retained the 1 item assessing lack of satiety (TFEQ39), which accounted for variance in the gap in the high range of pathology. Second, we retained 1 of 2 items assessing the domain of preoccupation of food. We retained the item that accounted for variance at the middle range of pathology (FCQT10). Third, we retained 1 item assessing the domain of loss of control over eating that accounts for variance in gaps at the low and middle ranges of pathology (YFAS2), which accounted for variance at the lower and middle ranges of pathology. Finally, we retained an additional item that also assesses the domain of loss of control over eating that accounts for variance at the lowest range of pathology (DEBQ2). The resulting 13 items comprised the Reward-based Eating Drive Scale - Revised (RED-13).

#### Confirmatory Factor Analysis (CFA)

For comparison with other studies, we first computed 1-factor and 3-factor CFAs using the RED-13 scale (1-factor model: X^2^ = 538.051, df = 65, *p* < 0.001, CFI = 0.967, RMSEA = 0.145, SRMR = 0.076; 3-factor model: X^2^ = 179.844, df = 62, *p* < 0.001, CFI = 0.992, RMSEA = 0.074, SRMR = 0.042; factor correlations: *r* = 0.76–0.88).

We then computed these CFAs using the RED-13 scale in the Study 3 sample (1-factor model: X^2^ = 471.857, df = 65, *p* < 0.001, CFI = 0.974, RMSEA = 0.134, SRMR = 0.071; 3-factor model X^2^ = 185.029, df = 62, *p* < 0.001, CFI = 0.992, RMSEA = 0.076, SRMR = 0.042; factor correlations: *r* = 0.78–0.92; **Supplementary Figure S4**). Analyses using data from each Study 3 and 4 suggest the 3-factor models better fit the data. Factor loadings of the RED-13 using each the Study 3 and 4 samples appear in **Table 3**.

#### Associations between RED-9, RED-13, and Additional Outcomes

Study 4 data indicated that BMI was positively associated with the RED-9 (*r* = 0.27, *p* < 0.001) and the RED-13 (*r* = 0.25, *p* < 0.001). Similarly, greater scores on the RED-9 (OR = 1.07, *b* = 0.07, se(*b*) = 0.03, *z* = 2.28, *p* = 0.031) and the RED-13 (OR = 1.06, *b* = 0.06, se(*b*) = 0.02, *z* = 2.4, *p* = 0.023) were each associated with greater odds of having type 2 diabetes. Last, greater scores on the RED-9 and the RED-13 were associated with greater craving for savory and for sweet (RED-9: savory: *r* = 0.45, < 0.001; sweet: *r* = 0.28 < 0.001; RED-13: *r* = 0.47, *p* < 0.001; sweet: *r* = 0.29, < 0.001).

## Discussion

The value of a scientific study hinges on the accuracy of measure that it employs. In this series of studies, we developed a revised 13-item version of the RED scale, which we have named the RED-13, in the service of more completely assessing the construct of RRE. In four large samples of adults, we first sought to ascertain if adding additional items to the original RED scale (RED-9; Epel et al., 2014) would improve the extent to which it assesses the full spectrum of RRE. As the RED-9 comprises both original items as well as items from existing measures, we sequentially considered different items from existing measures as potential additions to the RED-9 using IRT analyses (Baker, 2001; Partchev, 2004; Wirth and Edwards, 2007). The resultant RED-13 accounted for greater variability than the RED-9 by reducing gaps in assessment of RRE in middle-to-low ranges. We examined the psychometric properties of the resulting RED-13 in both Study 3 and 4 samples, and found that like the RED-9, the RED-13 was positively correlated with BMI and other relevant outcomes.

The RED-13 has many advantages over existing measures of eating behavior. A finding novel to this investigation is that in addition to BMI, the RED-13 was also related to self-reported diagnosis of type 2 diabetes as well as cravings for sweet and savory foods. Čukić et al. (2016) recently reported a positive association between eating-related impulsivity as indexed by a single item (“*when I am having my favorite food, I tend to eat too much*”) and a diabetes diagnosis, and current results further support and expand upon this association by employing a more complete assessment of control over eating (RED scale items). An association between the RED-13 and a diabetes diagnosis underscores the utility of the RRE construct to identify individuals at risk for poor metabolic health. Notably, the low number of diabetes cases warrants further testing of this association in larger samples.

The RED-13 is designed to capture three dimensions of the RRE construct: lack of control over eating, lack of satiety, and preoccupation with food. In all studies, a 3-factor model was a better fit for the data than a 1-factor model, suggesting that the scale indeed measures these three dimensions. Just as previous work has shown that reductions in RRE as assessed by the RED-9 can mediate the effects of obesity treatment on weight loss (Mason et al., 2016), the RED-13 may be a valuable tool for researchers interested in identifying and intervening upon these three modifiable behavioral risk factors in populations with type 2 diabetes.

Although other scales capture facets of RRE, the RED-13 is unique in that it captures a broader spectrum of RRE behavior. Existing validated measures of overeating behavior, such as the YFAS (Gearhardt et al., 2009) and the BES (Gormally et al., 1982), capture variability at the more severe end of RRE. The primary advantage of the RED-13 over the RED-9 is that it more completely accounts for variance in the tails of the RRE continuum. Specifically, the RED-13 captures more variance at lower levels of RRE, so it may be more sensitive to subtle changes in the RRE construct at this end of the continuum. For example, some individuals engage in passive overeating that results in gradual weight gain over time (Davis, 2013), and identifying small but incremental increases in RRE may play an important role in obesity phenotyping and treatment matching. For example, some individuals with obesity may experience greater reductions in food addiction symptoms when provided with a lifestyle intervention that includes self-regulation training in the form of mindfulness (relative to when they are just provided with a traditional lifestyle intervention; Mason et al., 2015b).

This series of studies has both strengths and weaknesses. Although Study 1 employed data collected in-person, Studies 2, 3, and 4 relied on Internet data collection using MTurk. MTurk is a popular and growing practice (Buhrmester et al., 2011), however, it suffers from certain limitations associated with Internet-based and self-report research (Goodman et al., 2013). For example, data from the latter three studies relied on self-reported weight, and the accuracy of such reporting depends on BMI status in a dose-dependent fashion: the larger the BMI, the less accurate the estimated body weight (Visscher et al., 2006). Hence, these data may provide a conservative estimate of the association between BMI and RED-13. Of note, the RED-9 was weakly correlated with BMI in Study 1, and this was likely due to the restricted BMI range for this sample (greater than 30.0 kg/m^2^). Thus, this analysis did not capture associations between the RED-9 and variability in the normal BMI range (18.5–24.9 kg/m^2^) or the overweight BMI range (25.0–29.9 kg/m^2^). Data for these analyses were cross-sectional and observational, thus precluding assertions about causality: That is, although greater RRE may lead to weight gain, it is also possible that excess adiposity could increase RRE via dysregulated appetite hormones and other metabolic abnormalities (Mietus-Snyder and Lustig, 2008). However, the original RED validation paper (Epel et al., 2014) reported longitudinal associations between RED-9 scores and weight gain over time. Major strengths of this series of studies include rigorous statistical methodology (IRT) that allowed us to systematically optimize the original RED-9 scale and sample items from a broad array of existing measures of eating behavior. Additionally, we capitalized on existing data (Studies 1 and 2) and collected original data (Studies 3 and 4), which each included relatively large samples. Between the original series of validation studies and those in this series of studies, 2,120 respondents have been involved in the creation of the RED-9 and the RED-13.

In sum, the RED-13 scale is a brief and psychometrically sound scale that captures variability across the spectrum of RRE. The RED-13 is positively associated with BMI, food cravings, and self-reported type 2 diabetes diagnosis. Researchers and clinicians may find the RED-13 useful to identifying individuals who engage in RRE, defined as comprising a lack of control over eating, lack of satiety, and preoccupation with food. Higher scores on the RED-13 may portend poor metabolic health. Identifying RRE in the middle and lower ranges of the spectrum may be one avenue by which to stem the tide of the growing obesity epidemic: Identifying individuals at risk for weight gain over time may be a promising initial step toward intervening on this trajectory.

## Ethics Statement

These studies were carried out in accordance with the recommendations of the University of California, San Francisco (UCSF) Institutional Review Board (IRB) with documented informed consent from all participants, who provided in-person written informed consent (Study 1; in person) or electronic informed consent (Studies 2, 3, and 4) in accordance with the Declaration of Helsinki. Protocols were approved by the UCSF IRB.

## Author Contributions

AM wrote the first draft of all manuscript components excepting the figures, and collected data for Study 4. UV conducted statistical analyses and created all figures. MA collected, collated, and organized data for Studies 1 and 2 and consulted on statistical analyses. AT collected data for Study 3 and provided feedback on the manuscript, including generation of original text for several areas. AD contributed to manuscript preparation and editing. EE collected data for Studies 1 and 2 and provided feedback on the manuscript. FH collected data for Studies 1 and 4 and provided feedback on the manuscript.

## Conflict of Interest Statement

The authors declare that the research was conducted in the absence of any commercial or financial relationships that could be construed as a potential conflict of interest.

## Supplementary Material

**FIGURE S1 | Study 1: Person-item map and histogram depicting thresholds on the spectrum of reward-related eating (RRE) using all items with factor loadings greater than 0.45 (*n* = 27 additional items, plus all RED-9 items).** The histogram at top displays the locations of participants on the RRE construct. The top row of the person-item map at bottom depicts the locations of the gaps in coverage of the RRE construct left by the RED-9. The 27 items listed below “thresholds of RED items” are ordered by the average of their thresholds’ values, and colored by the respective scale from which they originate. Circles depict each threshold, i.e., location on the RRE construct where people are most likely to move from one response option to the next. Gray rectangles appear whenever the gap between two current RED item thresholds is wider than 0.29 units.

**FIGURE S2 | Study 2: Person-item map and histogram depicting thresholds on the spectrum of RRE using all items with factor loadings greater than 0.45 (*n* = 77 additional items, plus all RED-9 items).** See **Supplementary Figure S1** note.

**FIGURE S3 | Study 3: Person-item map and histogram depicting thresholds on the spectrum of RRE using all items retained from Studies 1 and 2 (*n* = 32 additional items, plus all RED-9 items).** See **Supplementary Figure S1** note.

**FIGURE S4 | Study 4: Person-item map and histogram depicting thresholds on the spectrum of RRE applying the item set identified in Study 4 (*n* = 9 additional items, plus all RED-9 items) to the dataset from Study 3.** See **Supplementary Figure S1** note. Bars in darker gray indicate gaps in RRE coverage left by the RED-13. Bars in lighter gray indicate gaps in coverage left by RED-9.
