# Supplementary figures and images for: Improving Assessment of the Spectrum of Reward-Related Eating: The RED-13
Source: Front Psychol. 2017 May 30;8:795. doi: 10.3389/fpsyg.2017.00795 (PMC5447741; doi:10.3389/fpsyg.2017.00795)

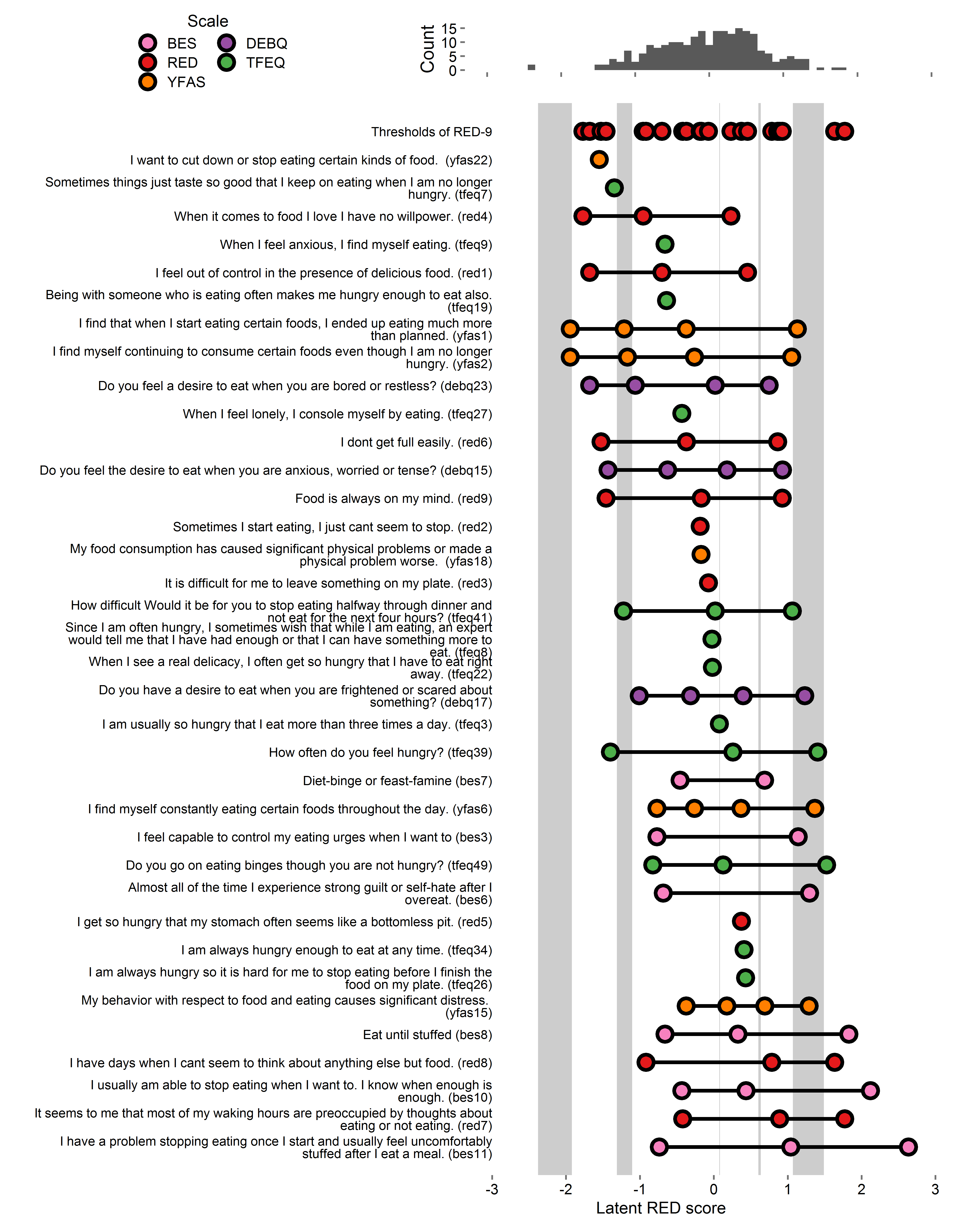

Supplement: Supplementary file 1 [file Image_1.tiff]

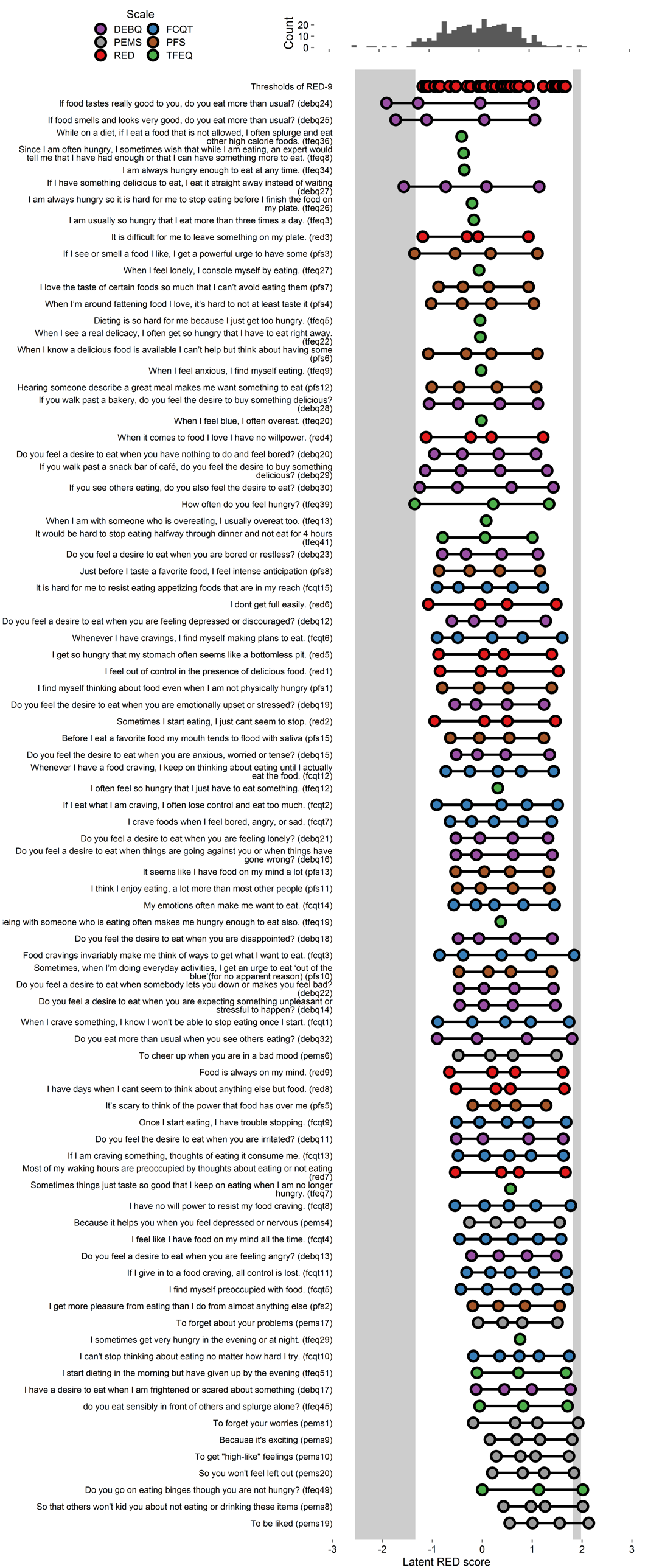

Supplement: Supplementary file 2 [file Image_2.tiff]

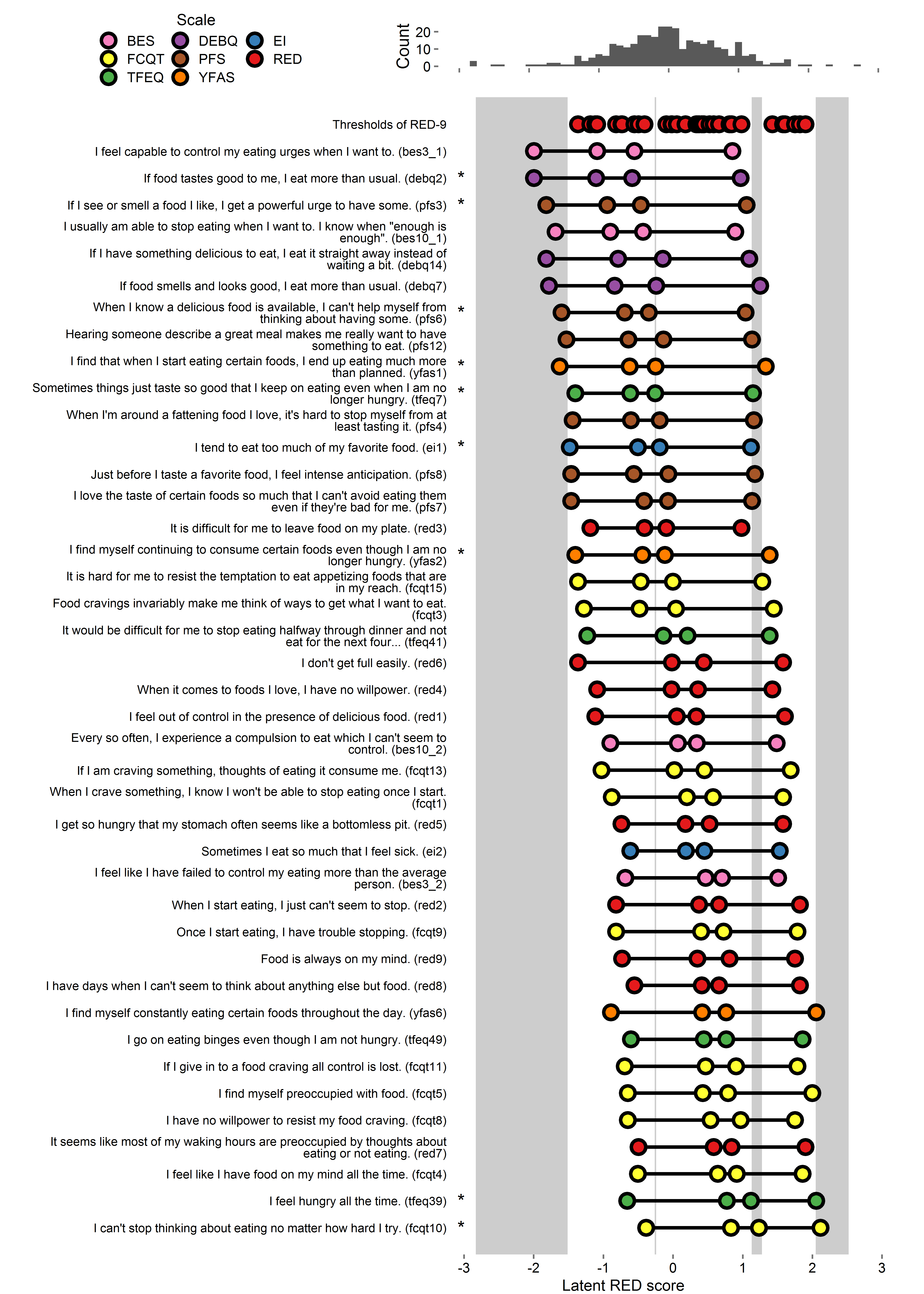

Supplement: Supplementary file 3 [file Image_3.tiff]

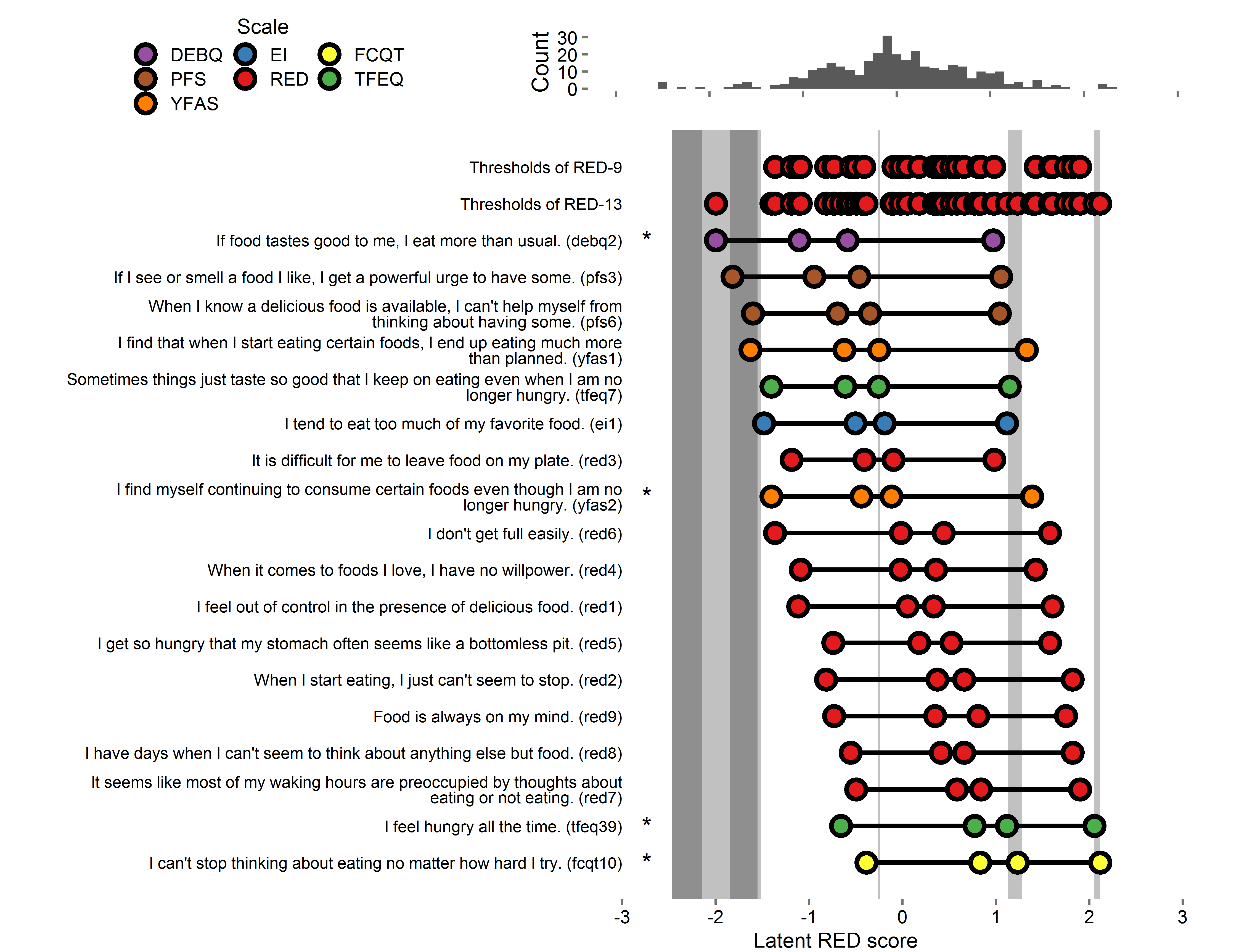

Supplement: Supplementary file 4 [file Image_4.tiff]
